# Serum lipid profiles among patients initiating ritonavir-boosted atazanavir versus efavirenz-based regimens

**DOI:** 10.1186/1742-6405-6-13

**Published:** 2009-06-22

**Authors:** Anuradha Ganesan, Lorie Benning, Elizabeth T Golub, Mark Riddle, Nancy Crum-Cianflone, Sybil Tasker, Lisa Jacobson, Stephen J Gange

**Affiliations:** 1 Infectious Disease Clinical Research Program, Uniformed Services University of the Health Sciences, National Naval Medical Center, Bethesda, Maryland, USA; 2 Department of Epidemiology, Johns Hopkins Bloomberg School of Public Health, Baltimore, Maryland, USA; 3 Naval Medical Research Center, Silver Spring, Maryland, USA; 4 Infectious Disease Clinical Research Program, Uniformed Services University of the Health Sciences, Naval Medical Center San-Diego, Calfornia, USA

## Abstract

**Background:**

Antiretrovirals used to treat HIV-infected patients have the potential to adversely affect serum lipid profiles and increase the risk of cardiovascular disease which is an emerging concern among HIV-infected patients. Since boosted atazanavir and efavirenz are both considered preferred antiretrovirals a head to head comparison of their effects on serum lipids is needed.

**Aim:**

The primary objective of the study was to compare the effects of atazanavir (boosted and unboosted) and efavirenz based regimens on serum lipid profiles.

**Study Design:**

Prospective cohort study nested within three ongoing cohorts of HIV-infected individuals.

**Study Population and Methods:**

Participants initiating either atazanavir or efavirenz based regimens with documented pre- and post-initiation lipid values. Multivariate linear regression was conducted to estimate adjusted mean differences between treatment groups for high density lipoprotein cholesterol (HDL-c), non-HDL-c, and log total cholesterol (TC) to HDL-c ratio outcomes; log-linear regression models were used to estimate differences in prevalence of low HDL-c and desirable TC.

**Results:**

The final study population was comprised of 380 efavirenz and 281 atazanavir initiators. Both atazanavir and efavirenz users had increases in serum HDL-c and decreases in TC/HDL ratio. In comparison to individuals initiating efavirenz, boosted atazanavir users on average had lower HDL-c (-4.12 mg/dl, p < 0.001) and non HDL-c (-5.75 mg/dl, p < 0.01), but similar declines in TC/HDL ratio.

**Conclusion:**

Both efavirenz and atazanavir-based regimens (boosted and unboosted) resulted in similar beneficial declines in the TC/HDL ratio.

## Background

Use of antiretrovirals can adversely affect serum lipid levels and contribute to cardiovascular risk, an emerging concern among HIV-infected patients. [1,2] Therefore, careful comparisons of the effects of commonly used antiretrovirals on serum lipids are needed. Efavirenz (EFV) and atazanavir (TAZ) are two commonly used antiretrovirals. The results of a phase 3 study comparing EFV and TAZ based regimens demonstrate similar virologic efficacy with the use of either agent, though EFV use resulted in dyslipidemia while TAZ use did not. [3] However, the current treatment paradigm favors the use of TAZ in combination with low dose ritonavir (boosted TAZ) and not unboosted TAZ.[4] Since, the addition of ritonavir to TAZ results in dyslipidemia [5], there is a need to compare and contrast the magnitude of the effects of boosted TAZ and EFV based regimens on serum lipids. To this end, we utilized data from a racially diverse group of HIV-infected individuals with the specific aims of comparing the effects of boosted TAZ and EFV on serum lipids. In this study we observed beneficial changes, namely declines in total cholesterol (TC)/high density lipoprotein cholesterol (HDL) ratio and increases in HDL-c, in serum lipids with all three regimens (unboosted TAZ, boosted TAZ, and EFV)

## Methods

### Study population

Data were collected from participants enrolled in three independent ongoing prospective cohort studies: the Multicenter AIDS Cohort Study (MACS), the Women's Interagency HIV Study (WIHS), and United States Navy beneficiaries followed at two of three Navy HIV Evaluation and Treatment Units. Recruitment and follow-up procedures for all three patient groups have been previously described. [6-8] Eligible subjects initiated regimens of two or more nucleoside reverse transcriptase inhibitors (NRTI) with either 1) EFV or 2) TAZ or boosted TAZ, using these criteria 1,020 EFV and 655 TAZ initiators were identified. Excluding initiators who did not have lipids measured both pre and post initiation (430 EFV and 108 TAZ), those missing information on antiretroviral use during the study period (41 EFV and 50 TAZ), those using both agents at the pre visit (137 EFV and 179 TAZ), or using lipid lowering therapy at the pre or first post visit (32 EFV and 37 TAZ), 380 EFV and 281 TAZ initiators comprised our final study population. Complete antiretroviral history including start and stop dates were determined from chart review, electronic pharmacy records, or self-report assessed using questionnaires and photo cards. Individuals were followed up to the first occurrence of 1) discontinuation of EFV or TAZ (29% EFV, 30% TAZ), 2) initiation of lipid-lowering therapy (4% EFV, 1% TAZ), or 3) last lipid measurement within two years of initiating EFV/TAZ (67% EFV, 68% TAZ). There was no difference in censoring between the two ARV groups (p = 0.21). EFV or TAZ was used <1 to 6 months prior to the first post visit for 76% of participants, 6–12 months for 19%, and 12–24 months for 5%.

### Outcome ascertainment

MACS began routine measurement of serum lipids for all participants in April 1999; WIHS began in April 2004. Baseline levels were measured retrospectively for selected MACS participants, and for all WIHS participants seen between October 2001 and March 2004. Navy participants had lipids measured at all visits. Both MACS and WIHS used a central laboratory, while the Navy participants used their hospital laboratories for lipid measures. Three continuous and two binary outcomes based on the updated National Cholesterol Education Program (NCEP) ATP III Guidelines [9] were defined: 1) high-density lipoprotein cholesterol (HDL-c measured in mg/dL), 2) non-HDL-c (mg/dL) calculated as total cholesterol (TC) minus HDL-c, 3) natural log-transformed TC to HDL-c ratio, 4) low HDL-c (<40 vs. ≥ 40 mg/dL), and 5) desirable TC (<200 vs. ≥ 200 mg/dL).

### Statistical methods

Multivariate linear regression was used to estimate adjusted mean differences between treatment groups for HDL-c, non-HDL-c, and log TC to HDL-c ratio; multivariate log-linear regression was used to estimate prevalence ratios for low HDL-c and desirable TC. For each outcome, two models with different potential confounders were included. Model 1 adjusted for sex, race/ethnicity, age at EFV/TAZ initiation, history of diabetes mellitus or thyroid dysfunction, baseline hepatitis C virus antibody status, pre-initiation HDL-c and non-HDL-c, CD4+ cell count, HIV-1 viral load, pre-initiation ART- and class- (NNRTI/PI) naïve status, years of HAART exposure and follow up time. In model 2, we further adjusted for post-initiation CD4+ count and HIV-1 viral load and type of NRTI backbone. Two potential a priori effect modifiers of regimen with dyslipidemia outcomes were also investigated: (a) follow-up time (time elapsed between the date of EFV/TAZ initiation and the date lipids were collected) and (b) race/ethnicity. We specifically evaluated the role of race and ethnicity, given recent reports that suggest the effects of antiretrovirals on serum lipids are modified by host characteristics including race. [10]

For each model, generalized estimating equation methods (GEE) were used to account for within-person correlation of repeated measurements. Data were complete for all cofactors for 357 (94%) of EFV and 259 (94%) of TAZ users; (single-chain) Markov-chain Monte Carlo multiple-imputation methods were used to complete missing covariate data. [11]

## Results

The study population consisted of 380 EFV initiators and 281 TAZ initiators (79% of whose regimens were boosted), racial minorities (47% African-Americans and 16% Hispanics) and women (48%) were well represented. Table 1 provides the baseline characteristics of the two groups. Adjusted for baseline CD4 counts, follow-up time, regimen type, and sex, CD4+ cell counts increased on average by 57 cells per year. The proportion of individuals having an undetectable viral load (i.e. < 80 copies/ml) increased from 18% to 63% among all subjects. At any point in time, on average, TAZ users had lower CD4+ cell count (-26, p = 0.05) and lower odds of an undetectable viral load (0.60, p < 0.0001).

**Table 1:** Baseline characteristics of 380 efavirenz and 281 atazanavir initiators.

Patient Characteristics*	Efavirenz initiators (N = 380)	Atazanavir initiators (N = 281)	P-value**
**Study site**			<0.0001

Navy	32%	25%	

MACS	33%	15%	

WIHS	36%	60%	

			

Male	63%	38%	<0.0001

			

**Race/Ethnicity**			<0.0001

Caucasian (Hispanic & Non Hispanic)	43%	28%	

African-American (Hispanic & Non-Hispanic)	46%	49%	

Hispanic (non-white, non-black) & other	11%	22%	

			

Age at switch/initiation	41 (34, 47)	41 (35, 46)	0.91

			

**Medical History**			

Diabetes Mellitus	11%	17%	0.03

Thyroid Disease	3%	6%	0.07

Hepatitis C antibody status	15%	19%	0.18

			

**Treatment Characteristics**			

HAART initiation date	Aug 2001 (Aug 97-Sep03)	Dec 1998 (May 97-Sep 02)	0.003

ART-naïve at initiation	34%	12%	<0.0001

Class-naïve at initiation	80%	30%	<0.0001

Years of HAART exposure at initiation	0.5 (0.1, 2.9)	3.6 (1.2, 5.7)	<0.0001

Atazanavir regimen included Ritonavir		79%	

			

**HIV biomarkers prior to initiation**			

CD4+ cell count	332 (216, 464)	289 (202, 462)	0.11

Log_10 _HIV RNA	4.15 (2.65, 4.75)	4.01 (2.59,4.76)	0.42

Viral load <80 copies/mL	19%	17%	0.50

			

Post-initiation HIV characteristics***			

CD4+ cell count	434 (303, 581)	410 (241, 575)	0.02

Log_10 _HIV RNA	1.90 (1.90, 2.38)	1.90 (1.90, 2.51)	<0.0001

Viral load <80 copies/mL	68%	58%	0.0003

NRTI backbone included			

TDF or ABC & D4T, DDI, or AZT	19%	28%	0.0003

TDF or ABC but not D4T, DDI, or AZT	32%	58%	<0.0001

D4T, DDI, or AZT but not TDF or ABC	49%	14%	<0.0001

* Median (IQR) for continuous characteristics ** From chi-square tests (categorical) or Wilcoxon rank sum test (continuous) *** Collapsed across all follow-up

The first two columns of Table 2 show the mean or prevalence of each of the outcomes at the baseline (pre-initiation) and post-initiation visits. The subsequent columns of Table 2 show the differences in post-initiation markers relative to those initiating EFV. There were no differences in the baseline TC, non HDL-c, or HDL-c among EFV and TAZ users. After adjusting for potential confounders, both boosted and unboosted TAZ initiators showed a smaller increase in HDL-c than EFV initiators. Those initiating boosted (but not unboosted) TAZ also showed lower non-HDL-c as compared to EFV initiators. All three regimens resulted in similar declines in TC/HDL ratios. The proportion of subjects who met study specified criteria for low HDL-c declined with all three regimens and was not statistically different among the three groups. Subjects meeting study specified criteria for desirable cholesterol were similar across treatment groups. The interaction between regimen type and follow-up time was not significant in any model.

**Table 2:** Comparison of the changes in serum lipids following initiation of either Efavirenz or Atazanavir based regimens.

		Mean						
Continuous Lipid Outcome	ARV Group	Pre	Post	Unadjusted Post Difference	Model 1* Adjusted Difference	p-value	Model 2** Adjusted Difference	p-value
**HDL-c (mg/dL)**	EFV	39	46	Ref	Ref		Ref	
	Unboosted TAZ	39	42	-4	-3.92	0.003	-3.43	0.008
	Boosted TAZ	38	41	-5	-4.12	<0.001	-4.00	<0.001
**Non- HDL-c (mg/dL)**	EFV	131	136	Ref	Ref		Ref	
	Unboosted TAZ	117	131	-5	-1.92	0.55	-1.08	0.73
	Boosted TAZ	126	125	-11	-5.75	0.01	-4.57	0.04
**TC/HDL**	EFV	4.45	4.02	Ref	Ref		Ref	
	Unboosted TAZ	4.45	4.10	1.99	4.41	0.14	3.94	0.19
	Boosted TAZ	4.10	3.97	-1.24	2.62	0.21	3.40	0.11

								

		Prevalence						
Binary Lipid Outcome	ARV Group	Pre	Post	Unadjusted Post Prevalence Ratio	Model 1* Adjusted Prevalence Ratio	p-value	Model 2** Adjusted Difference	p-value

**HDL<40 (mg/dL)**	EFV	52%	33%	Ref	Ref		Ref	
	Unboosted TAZ	55%	39%	1.18	1.05	0.79	1.01	0.95
	Boosted TAZ	53%	42%	1.27	1.18	0.18	1.19	0.17
**TC<200 (mg/dL)**	EFV	74%	62%	Ref	Ref			
	Unboosted TAZ	85%	73%	1.18	1.07	0.59	1.05	0.71
	Boosted TAZ	81%	78%	1.26	1.16	0.09	1.13	0.16

* Adjusted for sex, race, age, history of chronic diseases, pre-initiation HDL and non-HDL cholesterol, CD4, HIV RNA, therapy, and follow-up time

** In addition to variables above, also adjusted for post-initiation CD4, HIV RNA, and type of NRTI backbone

Changes in serum lipids varied by race; in comparison to Caucasians, mean post-initiation HDL-c was significantly higher (+5 mg/dL, p < 0.0001) among African-Americans, while Hispanics had significantly lower mean non-HDL-c (-5 mg/dl, p = 0.04). In African-Americans, the TC/HDL ratio and proportion who met study specified criteria for low HDL-c were significantly lower by 8% (p < 0.0001) and 34% (p = 0.0001) respectively. The TC/HDL was also significantly lower in Hispanics by 5% (p = 0.03). However, the interaction between regimen type and race was not significant in any model.

In a separate analysis to assess possible bias resulting from excluding individuals who had post-initiation lipids but were missing pre-initiation measures, we ran additional multivariate linear regression models using GEE methods for HDL-c and non-HDL-c, adjusting for sex, race, age, and follow-up time, first excluding those who were missing pre-initiation measures (MPM) and then including them. When the MPM individuals were excluded, TAZ users had 5.97 mg/dL lower HDL-c than EFV users (p < 0.001); when they were included, TAZ users had 6.48 mg/dL lower HDL-c (p < 0.001). For non-HDL-c, the differences were -7.46 mg/dL (p = 0.046) excluding MPM individuals and -13.86 mg/dL (p < 0.001) including them

## Discussion

The results of our study are similar to those observed in a randomized clinical trial comparing EFV and unboosted TAZ in naïve patients.[3] In this study, those initiating EFV had greater increases in TC and HDL-c. Our results would suggest that the addition of low dose ritonavir to TAZ appears not to negate these effects. Interestingly, the use of all three regimens (EFV, boosted and unboosted TAZ) were associated with beneficial changes in serum lipids in comparison to baseline namely increases in HDL-c, and decreases in TC/HDL ratio. In addition, the proportion of patients who switched HDL-categories (low to normal) increased with all three regimens. However, in comparison to boosted TAZ-users, EFV-users had greater increases in both HDL and non-HDL-c. Whether differences in the class of lipids preferentially affected by these agents will influence the risk of future coronary artery disease is unknown and warrants study.

Given the racial diversity of the study population we were also able to evaluate the effects of these agents by race. African-Americans and Hispanics demonstrated a less atherogenic lipid profile in response to therapy with either agent. It's unlikely that lifestyle factors alone account for the differences we observed; genetic variations probably played a role. Differences in host genetic characteristics are known to influence levels of both EFV and TAZ and serum lipid levels independently. [12,13] Increases in HDL-c and non HDL-c in response to ART are associated with polymorphisms in the cholesterol ester transfer protein and multi-drug resistance genes. [14] Thus far, pharmacogenetic studies have been conducted in racially homogeneous populations; futures studies should include racially representative population, to help explain these differences. [14,15]

One possible criticism regarding our study is its observational design with the potential for selection bias and confounding by unmeasured variables. As described previously, we conducted a sub-group analysis in patients with missing pre-initiation lipid measures. Similar results were observed among those with and without pre-initiation measures, thereby indicating that a selection bias was probably not operational and further validating our results.

## Conclusion

In conclusion, clinicians treating HIV-infected patients can be reassured that the use of both boosted TAZ and EFV-based regimens results in a favorable lipid profile, as measured by changes in serum TC/HDL ratio. However, since subtle metabolic effects are likely to become increasingly important in decisions regarding optimal drug therapy in HIV-infected patients, future studies should explore the effects of drugs on lipoprotein subclasses and the effects of host genetics on metabolic profiles. [16]

## Competing interests

The authors declare that they have no competing interests.

## Authors' contributions

AG was responsible for the overall conduct of the study including conception and design of the study, data acquisition, data analysis, and drafting of the manuscript. LB was responsible for data analysis, drafting and critical review of the manuscript. ETG participated in the study design and the critical review of the manuscript. MR participated in the data analysis and the critical review of the manuscript. NCC participated in the data collection and the critical review of the manuscript. ST participated in the study design and the critical review of the manuscript. LJ participated in the study design and the critical review of the manuscript. SG participated in the study design, data analysis, drafting and critical review of the manuscript.

## References

[B1] Friis-Møller N, Reiss P, Sabin CA, Weber R, Monforte A, El-Sadr W, Thiébaut R, De Wit S, Kirk O, Fontas E, Law MG, Phillips A, Lundgren JD, DAD Study Group (2007). Class of antiretroviral drugs and the risk of myocardial infarction. N Engl J Med.

[B2] Fontas E, van Leth F, Sabin CA, Friis-Møller N, Rickenbach M, d'Arminio Monforte A, Kirk O, Dupon M, Morfeldt L, Mateu S, Petoumenos K, El-Sadr W, de Wit S, Lundgren JD, Pradier C, Reiss P, D:A:D Study Group (2004). Lipid profiles in HIV-infected patients receiving combination antiretroviral therapy: are different antiretroviral drugs associated with different lipid profiles?. J Infect Dis.

[B3] Squires K, Lazzarin A, Gatell JM, Powderly WG, Pokrovskiy V, Delfraissy JF, Jemsek J, Rivero A, Rozenbaum W, Schrader S, Sension M, Vibhagool A, Thiry A, Giordano M (2004). Comparison of once-daily atazanavir with efavirenz, each in combination with fixed-dose zidovudine and lamivudine, as initial therapy for patients infected with HIV. J Acquir Immune Defic Syndr.

[B4] (2006). Guidelines for the use of antiretroviral agents in HIV-infected adults and adolescents.

[B5] Niel Malan DR, Krantz E, David N, Wirtz V, Hammond J, McGrath D (2008). Efficacy and Safety of Atazanavir, With or Without Ritonavir, as Part of Once-Daily Highly Active Antiretroviral Therapy Regimens in Antiretroviral-Naive Patients. J Acquir Immune Defic Syndr.

[B6] Kaslow RA, Ostrow DG, Detels R, Phair JP, Polk BF, Rinaldo CR (1987). The Multicenter AIDS Cohort Study: rationale, organization, and selected characteristics of the participants. Am J Epidemiol.

[B7] Barkan SE, Melnick SL, Preston-Martin S, Weber K, Kalish LA, Miotti P, Young M, Greenblatt R, Sacks H, Feldman J (1998). The Women's Interagency HIV Study. WIHS Collaborative Study Group. Epidemiology.

[B8] Crum NF, Riffenburgh RH, Wegner S, Agan BK, Tasker SA, Spooner KM, Armstrong AW, Fraser S, Wallace MR, Triservice AIDS Clinical Consortium (2006). Comparisons of causes of death and mortality rates among HIV-infected persons: analysis of the pre-, early, and late HAART (highly active antiretroviral therapy) eras. J Acquir Immune Defic Syndr.

[B9] Third Report of the National Cholesterol Education Program (NCEP) (2002). Expert Panel on Detection, Evaluation, and Treatment of High Blood Cholesterol in Adults (Adult Treatment Panel III) final report. Circulation.

[B10] Foulkes AS, Wohl DA, Frank I, Puleo E, Restine S, Wolfe ML, Dube MP, Tebas P, Reilly MP (2006). Associations among race/ethnicity, ApoC-III genotypes, and lipids in HIV-1-infected individuals on antiretroviral therapy. PLoS Med.

[B11] Schafer J (1997). Analysis of Incomplete Multivariate Data.

[B12] Rotger M, Tegude H, Colombo S, Cavassini M, Furrer H, Décosterd L, Blievernicht J, Saussele T, Günthard HF, Schwab M, Eichelbaum M, Telenti A, Zanger UM (2007). Predictive value of known and novel alleles of CYP2B6 for efavirenz plasma concentrations in HIV-infected individuals. Clin Pharmacol Ther.

[B13] Rodríguez-Nóvoa S, Martín-Carbonero L, Barreiro P, González-Pardo G, Jiménez-Nácher I, González-Lahoz J, Soriano V (2007). Genetic factors influencing atazanavir plasma concentrations and the risk of severe hyperbilirubinemia. Aids.

[B14] Arnedo M, Taffé P, Sahli R, Furrer H, Hirschel B, Elzi L, Weber R, Vernazza P, Bernasconi E, Darioli R, Bergmann S, Beckmann JS, Telenti A, Tarr PE (2007). Swiss HIV Cohort Study Contribution of 20 single nucleotide polymorphisms of 13 genes to dyslipidemia associated with antiretroviral therapy. Pharmacogenet Genomics.

[B15] Alonso-Villaverde C, Coll B, Gómez F, Parra S, Camps J, Joven J, Masana L (2005). The efavirenz-induced increase in HDL-cholesterol is influenced by the multidrug resistance gene 1 C3435T polymorphism. Aids.

[B16] Duprez Daniel and the INSIGHT/SMART group (2009). High-Density lipoprotein particles but not Low-Density Lipoprotein Particles Predict Cardiovascular Disease Events in HIV Patients. Abstract Number 149.

